# No relevant cardiac, pharmacokinetic or safety interactions between roflumilast and inhaled formoterol in healthy subjects: an open-label, randomised, actively controlled study

**DOI:** 10.1186/1472-6904-11-7

**Published:** 2011-06-01

**Authors:** Christian de Mey, Nassr Nassr, Gezim Lahu

**Affiliations:** 1 ACPS - Applied Clinical Pharmacology Services, Mainz-Kastel, Germany; 2 Nycomed GmbH, Konstanz, Germany

## Abstract

**Background:**

Roflumilast is an oral, selective phosphodiesterase 4 inhibitor with anti-inflammatory effects in chronic obstructive pulmonary disease (COPD). The addition of roflumilast to long-acting bronchodilators improves lung function in patients with moderate-to-severe COPD. The present study investigated drug-drug interaction effects between inhaled formoterol and oral roflumilast.

**Methods:**

This was a single-centre (investigational clinic), open, randomised, multiple-dose, parallel-group study. In Regimen A, healthy men were treated with roflumilast (500 μg tablet once daily; Day 2-18) and concomitant formoterol (24 μg twice daily; Day 12-18). In Regimen B, healthy men were treated with formoterol (24 μg twice daily; Day 2-18) and concomitant roflumilast (500 μg once daily; Day 9-18). Steady-state plasma pharmacokinetics of roflumilast, roflumilast N-oxide and/or formoterol (C_max _and AUC_0-τ_) as well as pharmacodynamics - blood pressure, transthoracic impedance cardiography (ZCG), 12-lead digital electrocardiography, peripheral blood eosinophils, and serum glucose and potassium concentrations - were evaluated through Day 1 (baseline), Day 8 (Regimen B: formoterol alone) or Day 11 (Regimen A: roflumilast alone), and Day 18 (Regimen A and B: roflumilast plus formoterol). Blood and urine samples were taken for safety assessment at screening, pharmacokinetic profiling days and Day 19. Adverse events were monitored throughout the study.

**Results:**

Of the 27 subjects enrolled, 24 were evaluable (12 in each regimen). No relevant pharmacokinetic interactions occurred. Neither roflumilast nor formoterol were associated with significant changes in cardiovascular parameters as measured by ZCG, and these parameters were not affected during concomitant administration. Formoterol was associated with a slight increase in heart rate and a corresponding shortening of the QT interval, without changes in the heart rate-corrected QTc interval. There were small effects on the other pharmacodynamic assessments when roflumilast and formoterol were administered individually, but no interactions or safety concerns were seen after concomitant administration. No severe or serious adverse events were reported, and no adverse events led to premature study discontinuation.

**Conclusions:**

No clinically relevant pharmacokinetic or pharmacodynamic interactions were found when oral roflumilast was administered concomitantly with inhaled formoterol, including no effect on cardiac repolarisation. Roflumilast was well tolerated.

**Trial Registration:**

Clinicaltrials.gov NCT00940329

## Background

Roflumilast (3-cyclopropylmethoxy-N-(3,5-dichloropyridin-4-yl)-4-(difluoromethoxy)benzamide; CAS Registry number: 162401-32-3; molecular formula: C_17_H_14_Cl_2_F_2_N_2_O_3_) is a selective, oral, once-daily phosphodiesterase 4 (PDE4) inhibitor that has shown anti-inflammatory activity in pre-clinical studies [1-4], and in patients with chronic obstructive pulmonary disease (COPD) [5]. In large randomised clinical studies, roflumilast consistently improved lung function in patients with moderate-to-severe COPD [6], severe COPD [7], or severe airflow obstruction plus chronic bronchitis [8] compared with placebo.

Long-acting bronchodilators such as the β_2_-adrenoceptor agonists formoterol and salmeterol and the anticholinergic tiotropium [9-11] are central to the treatment of COPD; however, some patients have poor symptom control with these agents, particularly patients with more severe disease [12]. Two large clinical trials have investigated whether the addition of roflumilast improves lung function in patients with moderate-to-severe COPD who are already receiving long-acting bronchodilators [13]. In these trials, patients already receiving salmeterol or tiotropium were randomised to receive either oral roflumilast 500 μg or placebo once daily for 24 weeks, in addition to continued salmeterol or tiotropium treatment [13]. Compared with placebo, the addition of roflumilast improved mean pre-bronchodilator forced expiratory volume in both trials (p < 0.0001). Further, in a separate *in vitro *study, formoterol increased the inhibitory effect of roflumilast on cytokine and tumour necrosis factor-α production from human parenchymal and bronchial explants [14].

Both PDE inhibitors and β_2_-adrenoceptor agonists lead to an accumulation of intracellular cyclic adenosine monophosphate [15-17], which plays a key role in the regulation of cardiac function [18]. It is known that β_2_-adrenoceptor agonists are associated with adverse cardiac events [19]. In contrast, a previous study demonstrated that roflumilast had no significant effect on cardiac repolarisation (QT/QTc interval) in healthy subjects [20].

When administered as a single, oral 500 μg dose, roflumilast is readily and almost totally absorbed in healthy individuals, with a mean bioavailability of 79% [21] and dose-proportional pharmacokinetics observed within the 250-1000 μg dose range [22]. Repeated-dose pharmacokinetic profiles of roflumilast and its active metabolite roflumilast N-oxide have been well characterised, with median time to maximum plasma concentration (t_max_) values of 1 hour and 8 hours, respectively, and median effective plasma half-lives of 17 hours and 30 hours, respectively [22-24]. Roflumilast N-oxide has a PDE selectivity profile and potency *in vivo *similar to that of roflumilast, and a substantially (10-fold) greater area under the plasma concentration-time curve (AUC) [4,22]. It is therefore estimated to account for about 90% of the overall PDE4 inhibitory activity of roflumilast. To estimate the combined PDE4 inhibition of roflumilast and roflumilast N-oxide in humans following administration of roflumilast, the parameter termed 'total PDE4 inhibitory activity' (tPDE4i) has been established [23,25]. This parameter represents the sum of the overall exposure to roflumilast and roflumilast N-oxide by accounting for differences in intrinsic activity (IC_50_), free fraction (protein binding) and *in vivo *exposure (AUC values) of both compounds.

Roflumilast is metabolised to roflumilast N-oxide mainly through biotransformation via the cytochrome P450 (CYP) enzymes CYP3A4 and CYP1A2, and roflumilast N-oxide is cleared by CYP3A4. The CYP3A4 inhibitors erythromycin and ketoconazole have been shown to increase tPDE4i by 8-9% [26,27]; the CYP1A2 inhibitor fluvoxamine and the dual CYP3A4/1A2 inhibitors enoxacin and cimetidine increase tPDE4i by 59%, 25% and 47%, respectively [28-30]. Conversely, administration of the cytochrome P450 enzyme inducer rifampicin results in a reduction in tPDE4i by 58% [31].

Roflumilast and formoterol have a low potential for pharmacokinetic interaction because formoterol is eliminated mainly by direct glucuronidation and does not inhibit CYP isoenzymes at therapeutically relevant concentrations [32]. Following inhalation, formoterol is rapidly absorbed, with plasma concentrations increasing linearly with dose [32]. The kinetics of formoterol are similar after single and repeated administration, indicating no auto-induction or inhibition of metabolism [32]; however, there is a modest and self-limiting accumulation in plasma after repeated dosing in patients with COPD [32]. In a previous Phase I study, no apparent drug-drug interaction was found between roflumilast and orally co-administered formoterol (unpublished data; Nycomed GmbH, 2002). The nature and extent of a pharmacokinetic drug-drug interaction may, however, differ when formoterol is inhaled [33].

Since roflumilast is likely to be used concomitantly with a β_2_-adrenoceptor agonist in some patients, it is of interest to investigate whether and to what extent concomitant administration results in relevant pharmacokinetic and pharmacodynamic drug-drug interactions with a focus on cardiovascular effects.

## Methods

The protocol (Clinicaltrials.gov registration NCT00940329) was reviewed and approved by an independent ethics committee (Ethik-Kommission Landesärztekammer Rheinland-Pfalz Körperschaft des öffentlichen Rechts, Mainz, Germany) and competent health authorities (Bundesinstitut für Arzneimittel und Medizinprodukte [BfArM] Fachregistratur Klinische Prüfung, Bonn, Germany). The study was planned, conducted, analysed and reported in accordance with the principles of Good Clinical Practice, the Declaration of Helsinki and the provisions for the orderly conduct of clinical trials in the country of conduct.

### Subjects

Eligibility of subjects was evaluated on the basis of an extensive screening investigation performed within 3 weeks before admission to the study clinic, which included demography, medical history, review of co-medications, physical examination, recumbent blood pressure and pulse rate, electrocardiograms (ECGs), and laboratory safety tests (haematology, clinical chemistry, urinalysis, hepatitis and HIV serology).

Eligible subjects included Caucasian males aged 18 to 45 years with a body mass index between 18 and 30 kg/m^2^, and a body weight of > 50 kg. All subjects were willing and able to provide informed consent.

Exclusion criteria were as follows: previous participation in the study or in any other study; donation of blood or plasma within the last 30 days; presence of acute or chronic disease; presence of clinically relevant findings in the laboratory tests (including hepatitis and HIV serology and tests for alcohol and social drugs); signs or history of cardiac disease including QTc interval (Bazett's correction) ≥ 430 ms and PQ interval ≥ 220 ms; susceptibility to symptomatic orthostatic hypotension; previous gastrointestinal surgery other than appendectomy and herniotomy; use of any medication within the last 2 weeks or within less than 10 times the elimination half-life of the respective drug; history of any clinically relevant hypersensitivity (in particular to formoterol or other β_2_-adrenoceptor agonists, to roflumilast or to any inactive ingredient in the trial medication); smoking more than 10 cigarettes/day or equivalent; evidence or suspicion of alcohol or social drug abuse; excessive xanthine consumption; and any concern of lack of compliance or willingness to adhere to the study directives and restrictions.

### Interventions

This was an open-label, randomised, actively controlled, multiple-dose, parallel-group study with a fixed-administration sequence. Screening took place between Day -21 and Day -1; the study period lasted from Day -1 to Day 19; and the post-study examination was conducted within 1 week of the last intake of the study medication. Subjects were admitted to the clinic from the evening of Day -1 to the morning of Day 19. Subjects were instructed to avoid strenuous physical exercise from Day -3 until the post-study examination. Smokers were required to keep their smoking habits stable, but were not allowed to smoke during the main profiling days. Alcohol- and caffeine-containing beverages, as well as grapefruit juice, were not allowed from Day -2 to Day 19. In compliance with European guidelines, both drugs were given at the maximum recommended therapeutic dosage.

For Regimen A, subjects received 500 μg oral roflumilast once daily (daily dose: one tablet of roflumilast 500 μg) from the morning of Day 2 to the morning of Day 18. From the morning of Day 12 to the evening of Day 18 subjects also received 24 μg formoterol for oral inhalation delivered by a dry powder inhaler (DPI) device (Foradil^® ^P, Novartis Pharma GmbH, Vienna, Austria) twice daily, once in the morning and once in the evening (daily dose: 48 μg formoterol). For Regimen B, subjects received 24 μg formoterol for oral inhalation delivered by a DPI device (Foradil^® ^P) twice daily, once in the morning and once in the evening from the morning of Day 2 to the evening of Day 18. From the morning of Day 9 to the morning of Day 18 subjects also received 500 μg roflumilast once daily. The start of the concomitant phases for each regimen was dependent on the pharmacokinetic characteristics of roflumilast and formoterol; as more time is needed to achieve steady state for roflumilast than formoterol, the timings were different between Regimen A and Regimen B.

Roflumilast was taken orally with 240 mL plain water after an overnight fast and rest; after intake, a mouth check was performed for compliance control. No fluids were allowed within 2 hours after each dose on the profile days. On days when the two medications were co-administered, formoterol was administered within 1 minute of roflumilast being administered.

The main profiling days were scheduled on Days 1, 11 and 18 for Regimen A, and on Days 1, 8 and 18 for Regimen B. On these days, subjects continued their fast from the previous evening until 8 hours after morning dosing, and standardised meals were served at 8 hours (lunch) and 12.5 hours after morning dosing (dinner).

### Pharmacokinetic methods

Blood samples for pharmacokinetic assessments were taken on Days 11 and 18 for Regimen A and Days 8 and 18 for Regimen B, 30 minutes before dosing and 0.25, 0.5, 1, 2, 4, 6, 8, 10 and 12 hours after dosing, with additional samples taken at 14 and 24 hours for roflumilast (Days 11 and 18 for Regimen A, and Day 18 for Regimen B). Pre-dose blood samples for determination of trough levels were taken on Days 9 and 10 for Regimen A (morning only), and on Days 6 and 7 (morning and evening) for Regimen B. Blood samples (4.5 mL) were collected in heparinised tubes and plasma was obtained by centrifugation at 1550 g for 15 minutes. Plasma samples were stored at -20°C or below for roflumilast, and at -70°C for formoterol. Plasma concentrations of roflumilast and roflumilast N-oxide were determined using high-performance liquid chromatography coupled with tandem mass spectrometry (HPLC-MS/MS). Before the sample analysis we determined that formoterol did not interfere with the quantification of roflumilast or roflumilast N-oxide. The lower limit of quantitation (LLOQ) was 0.1 ng/L using a sample volume of 0.4 mL for both roflumilast and roflumilast N-oxide. The inter-day precision of this assay, as determined by the analysis of quality control (QC) samples, ranged from 5.3% to 10.0% for both analytes. The inter-day accuracy of the assay, as determined by the analysis of the QC samples, ranged from -6.8% to +2.5% for both analytes. Roflumilast and roflumilast N-oxide standard curves were valid up to 20 ng/mL and 40 ng/mL, respectively. Determination of roflumilast and roflumilast N-oxide was performed at Altana Pharma AG, Konstanz, Germany (Nycomed GmbH). Plasma concentrations of formoterol were determined using HPLC-MS/MS. The LLOQ in plasma was 0.4 pg/mL using a sample volume of 1.0 mL. Formoterol standard curves were valid up to 99.9 pg/mL. The intra-day precision for QC samples ranged from 0.5% to 13.1%. The intra-day accuracy of the formoterol QC samples ranged from -10.5% to +13.6%, the inter-day precision from 6.4% to 7.6%, and the inter-day accuracy from 0.7% to 7.1%. Determination of formoterol was performed at pharm-analyt Labor GmbH, Baden, Austria, under the supervision of Altana Pharma AG(Nycomed GmbH).

For roflumilast, roflumilast N-oxide and formoterol, the maximum plasma concentration (C_max_) was derived directly from the plasma concentrations. The AUC from time zero to the time of the last quantifiable concentration, which corresponded to the dosing interval of each analyte (AUC_0-τ_), was estimated using the linear trapezoidal method. Apparent clearance at steady state (CL/F; calculated by dose/AUC_τ_) was reported for roflumilast only. Pharmacokinetic variables were calculated by non-compartmental analysis using WinNonLin professional, version 4.01 (PharSight, Mountain View, California, USA). The calculation of tPDE4i was based on the equation described in Lahu et al. [27].

### Cardiovascular methods: blood pressure and transthoracic impedance cardiography

Cardiovascular effects were evaluated non-invasively by means of ECGs (cardiac rhythm, intra-cardiac conduction and ventricular repolarisation), oscillometric blood pressure and transthoracic impedance cardiography (ZCG; systolic time intervals, contractility indices and estimates of stroke volume and cardiac output). Cardiovascular assessments (oscillometric blood pressure, pulse rate, 12-lead ECG, and three-lead ZCG) were recorded before dosing and at 10, 20, 40 and 60 minutes, and at 2, 3, 4, 5, 6, 7 and 8 hours after morning dosing. Blood pressure, pulse rate and ECGs were also assessed at 12 hours after dosing, and on the morning of Day 19. All cardiovascular measurements were carried out after the subjects had been recumbent for at least 10 minutes; they generally stayed in bed from 1 hour before until 8 hours after morning dosing.

Systolic time intervals (STIs) and ZCG estimates of the systolic cardiac pump performance were derived from the simultaneous registration of a one-lead ECG, a phonocardiogram (PCG), and the rate of change of the transthoracic impedance (dZ/dt) to an AC current applied through the thoracic cage (CARDIODYNAGRAPH; Diefenbach GmbH, Wiesbaden, Germany). Tracings were captured and stored digitally during the study and subsequently analysed after the study by displaying the analogue ECG, PCG and ZCG signals on a computer screen. The relevant signal amplitudes and time intervals were delineated manually by operator-steered cursors; measurements were made for at least 10 artefact-free consecutive cardiac cycles [34,35]. From these tracings, the following variables were derived by direct measurement: RR interval (ms), total electromechanic systole (QS2 [ms]), ventricular ejection time (VET [ms]), baseline transthoracic impedance (Z0 [Ω]), and maximum negative velocity of transthoracic impedance changes during the cardiac cycle (dZ/dt_max _[Ω/s]). These variables were analysed for the 10 cycles and their means were used in the calculations of the following variables: heart rate (HR [bpm]), pre-ejection period (PEP [ms] = QS2 - VET), HR-corrected STIs (STI_c _and STI_i _according to Weissler et al. [36]), ZCG estimates of stroke volume (SV [mL] according to Kubicek's equation [37] using the equation of Geddes and Sadler for the specific resistance of blood [38]), cardiac output (CO [mL/min]), and total peripheral resistance (TPR [dyn.s.cm^-5^]). For the latter variable, the mean blood pressure (MBP [mmHg]) was used as calculated according to Wezler and Böger [39] from the systolic blood pressure (mmHg) and the diastolic blood pressure (mmHg).

ZCG analysis was carried out by a single analyst who was blinded with regard to subject, study regimen, profiling day within regimen, and time during the day. The time courses of ZCG variables were evaluated in two main data formats: untransformed (U, all profiling days) and time-matched for control Day 1 (δ, profiling days except control Day 1); the time courses of U- and δ-data were characterised by their morning pre-dose baseline values (BL_U _and BL_δ_) and the observed minimum (d_min_) and maximum (d_max_) values over the post-dosing time. Selected variables (HR, PEP, QS2, dZ/dt_max_, CO, and TPR) were compared by non-parametric estimates of the differences between the profiling days (point estimate and 95% confidence interval [CI] according to Hodges and Lehmann) [40].

### Electrocardiography

Digital 12-lead ECGs were recorded immediately after each ZCG recording. Tracings were analysed off-study by regimen-blinded qualified analysts, using previously reported methodology [20]. For each time point, three cycles were measured with regard to the RR, PQ and QT-intervals [ms]. The means of the three measurements were used for the calculation of HR and HR-corrected QTc intervals, according to Bazett's equation (QTcB = QT/RR^1/2^) [41] and Fridericia's equation (QTcF = QT/RR^1/3^) [41,42].

### Biological markers

During the main profiling days, blood was sampled at each ZCG time point for the determination of serum glucose and potassium concentrations. Samples were also collected 12 and 24 hours after morning dosing.

### Safety and tolerability

Blood and urine samples for conventional clinical laboratory safety tests were obtained at the screening visit, in the morning of each main profiling day, and on Day 19. Adverse events (AEs) were monitored throughout the study.

## Results

### Subjects

Twenty-seven healthy male subjects were enrolled and received the assigned investigational medication at least once (see Table 1 for demographic data). For Regimen A, 12 subjects were enrolled and all completed the study in accordance with the protocol specifications. For Regimen B, 15 subjects were enrolled: one withdrew consent on Day 11 and two were discontinued prematurely after erroneous dosing on Day 2, leaving 12 subjects who completed the study in accordance with the protocol.

**Table 1:** Subject demographics for the study population (all enrolled subjects)

Characteristic	Regimen A (n = 12)	Regimen B (n = 15)
Male, n (%)	12 (100)	15 (100)
Caucasian, n (%)	12 (100)	15 (100)
Age, years (median [range])	33 (25-44)	33 (21-44)
Body height, cm (median [range])	184 (169-192)	180 (168-185)
Body weight, kg (median [range])	83 (66-95)	75 (61-97)
Body mass index, kg/m^2 ^(median [range])	25 (23-28)	24 (21-30)
Smoking status, n		
Ex-smoker	2	4
Current	3	6
Never	7	5

### Pharmacokinetics

**Figure 1:**
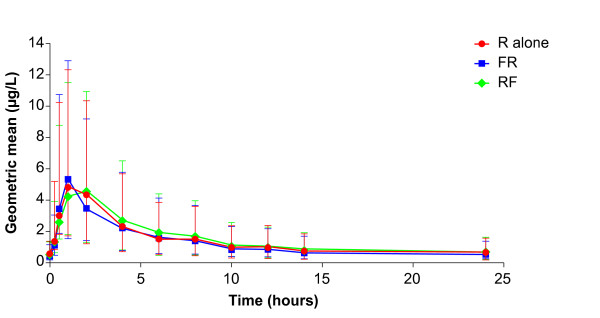
**Time course of the geometric mean (68% range) plasma concentrations of roflumilast (linear scale)**. R alone: roflumilast 500 μg once daily at steady state (Regimen A: Day 11; n = 12); RF: roflumilast 500 μg once daily and formoterol 24 μg twice daily at steady state (Regimen A: Day 18; n = 12); FR: formoterol 24 μg twice daily and roflumilast 500 μg once daily at steady state (Regimen B: Day 18; n = 12).

**Figure 2:**
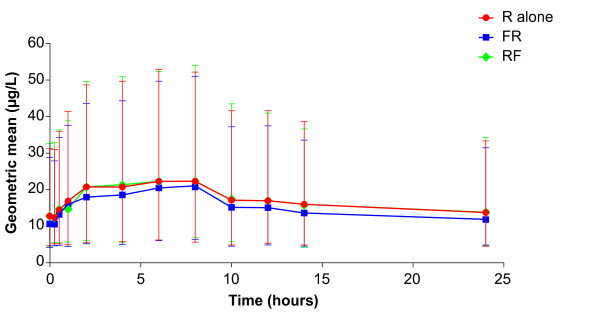
**Time course of the geometric mean (68% range) plasma concentrations of roflumilast N-oxide (linear scale)**. R alone: roflumilast 500 μg once daily at steady state (Regimen A: Day 11; n = 12); RF: roflumilast 500 μg once daily and formoterol 24 μg twice daily at steady state (Regimen A: Day 18; n = 12); FR: formoterol 24 μg twice daily and roflumilast 500 μg once daily at steady state (Regimen B: Day 18; n = 12).

**Figure 3:**
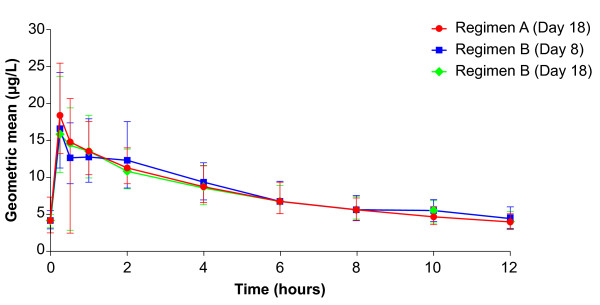
**Time course of the geometric mean (68% range) plasma concentrations of formoterol (linear scale)**. Regimen A: Day 18 (roflumilast 500 μg once daily and formoterol 24 μg twice daily at steady state; n = 12); Regimen B: Day 8 (formoterol 24 μg twice daily at steady state; n = 13) and Day 18 (formoterol 24 μg twice daily and roflumilast 500 μg once daily at steady state; n = 12).

The time courses of geometric mean plasma concentrations of roflumilast, roflumilast N-oxide and formoterol are shown in Figures 1, 2 and 3. Geometric means and their 68% ranges of steady-state pharmacokinetics of roflumilast, roflumilast N-oxide and formoterol are shown in Tables 2 and 3. There were no relevant changes in steady-state pharmacokinetics of roflumilast and roflumilast N-oxide when formoterol was added; similarly, there were no changes in steady-state pharmacokinetics of formoterol when roflumilast was added. There were some between-group effects, since steady-state AUC and C_max _values for roflumilast and roflumilast N-oxide were about 10-15% lower when roflumilast was co-administered with formoterol (Regimen B, Day 18), compared with roflumilast alone (Regimen A, Day 11) or roflumilast after addition of formoterol (Regimen A, Day 18).

**Table 2:** Geometric means (68% inter-percentile range) of the main pharmacokinetic variables for roflumilast and roflumilast N-oxide at steady state

	Regimen A		Regimen B
	**Day 11 (roflumilast alone)** **(n = 12)**	**Day 18 (roflumilast plus formoterol)** **(n = 12)**	**Day 18 (formoterol plus roflumilast)** **(n = 12)**

**Roflumilast**			
C _trough_ (μg/L)	0.53 (0.33-0.85)	0.50 (0.29-0.86)	0.37 (0.18-0.77)
C_max _(μg/L)	6.89 (4.94-9.62)	6.43 (4.79-8.63)	5.92 (4.27-8.22)
AUC_τ _(μg.h/L)	35.8 (27.8-46.1)	36.9 (28.1-48.4)	31.8 (21.2-47.7)
CL/F (L/h)	13.9 (10.8-17.9)	13.5 (10.3-17.7)	15.7 (10.4-23.5)

**Roflumilast N-oxide**			
C _trough_ (μg/L)	12.43 (8.24-18.76)	12.72 (8.18-19.79)	10.66 (6.29-18.06)
AUC_τ _(μg.h/L)	417 (299-582)	414 (293-584)	369 (254-537)
C_max _(μg/L)	23.3 (17.3-31.3)	23.7 (17.2-32.6)	22.04 (15.9-30.5)

AUC_τ_, area under the plasma concentration versus time curve; C_max_, maximum plasma concentration; C_trough_, trough plasma concentration; CL/F, apparent clearance at steady state.

**Table 3:** Geometric means (68% inter-percentile range) of the main pharmacokinetic variables for formoterol at steady state

	Regimen B		Regimen A
	**Day 8 (formoterol alone)** **(n = 13)**	**Day 18 (formoterol plus roflumilast)** **(n = 12)**	**Day 18 (roflumilast plus formoterol)** **(n = 12)**

**Formoterol**			
C_trough _(μg/L)	4.12 (3.05-5.57)	3.94 (3.13-4.96)	4.23 (2.43-7.38)
C_max _(μg/L)	16.7 (11.5-24.3)	17.2 (12.3-24.1)	18.7 (14.3-24.6)
AUC_τ _(μg.h/L)	96.7 (72.8-128)	93.1 (72.7-119)	93.2 (72.9-119)

AUC_τ_, area under the plasma concentration versus time curve; C_max_, maximum plasma concentration; C_trough_, trough plasma concentration.

### Impedance cardiography

#### Regimen A

The treatment medians for baseline Day 1, roflumilast monotherapy alone (R; Day 11), and concomitant roflumilast plus formoterol treatment (R+F; Day 18) are shown in Table 4 for untransformed morning pre-dosing values, untransformed d_max _and d_min _post-dosing values (including contrast of R vs D1), and time-matched d_max _and d_min _post-dosing values (including contrast of R+F vs R).

**Table 4:** Pharmacodynamic measures - Regimen A: treatment medians and treatment contrasts

Untransformed pre-dose morning values								
	**Median**			**Point estimate (95% CI)**				

**Variable**	**Day 1**	**Day 11 (R)**	**Day 18 (R+F)**	**Day 11-Day 1**	**Day 18-Day 11**		**Day 18-Day 1**	

HR (bpm)	59	63	61	3 (-5 to 8)	1 (-1 to 6)		5 (-2 to 10)	
PEP (ms)	106	94	95	-3 (-17 to 10)	-4 (-13 to 6)		-8 (-19 to 6)	
QS2 (ms)	435	423	430	-10 (-21 to 2)	-1 (-14 to 12)		-12 (-23 to 4)	
dZ/dt (Ω/s)	1.82	1.67	1.77	-0.06 (-0.29 to 0.16)	0.03 (-0.08 to 0.12)		-0.05 (-0.26 to 0.15)	
CO (L/min)	10.4	9.1	10.4	-0.6 (-1.7 to 0.5)	0.1 (-1.0 to 1.7)		-0.3 (-1.0 to 0.6)	
TPR (dyn.s.cm^-5^)	755	838	706	14 (-98 to 121)	-12 (-121 to 85)		-4 (-93 to 84)	

**Untransformed post-dose maximum and minimum values**								

	**d**_**max **_**(U)**				**d**_**min **_**(U)**			

	**Median**			**Point estimate (95% CI)**	**Median**			**Point estimate (95% CI)**

**Variable**	**Day 1**	**Day 11 (R)**	**Day 18 (R+F)**	**Day 11-Day 1**	**Day 1**	**Day 11 (R)**	**Day 18 (R+F)**	**Day 11-Day 1**

HR (bpm)	62	64	70	3 (-4 to 6)	52	54	61	4 (1 to 7)
PEP (ms)	119	121	114	3 (-7 to 11)	86	86	81	0 (-9 to 13)
QS2 (ms)	457	454	432	-1 (-11 to 11)	416	410	399	-9 (-21 to 8)
dZ/dt (Ω/s)	2.09	1.93	2.06	-0.05 (-0.25 to 0.15)	1.59	1.56	1.62	0.03 (-0.18 to 0.20)
CO (L/min)	10.3	10.6	11.7	0.2 (-1.1 to 1.4)	7.6	8.3	8.8	-0.4 (-1.2 to 0.4)
TPR (dyn.s.cm^-5^)	1013	934	877	47 (-124 to 178)	762	733	603	-18 (-96 to 43)

**Day 1-matched post-dose maximum and minimum values**								

	**d**_**max **_**(δ)**				**d**_**min **_**(δ)**			

	**Median**			**Point estimate (95% CI)**	**Median**			**Point estimate (95% CI)**

**Variable**		**Day 11 (R)**	**Day 18 (R+F)**	**Day 18-Day 11**		**Day 11 (R)**	**Day 18 (R+F)**	**Day 18-Day 11**

HR (bpm)		9	14	5 (-1 to 11)		-4	0	5 (1 to 9)
PEP (ms)		25	19	-8 (-19 to 4)		-27	-30	-2 (-11 to 10)
QS2 (ms)		19	4	-15 (-31 to -8)		-34	-47	-14 (-29 to 1)
dZ/dt (Ω/s)		0.28	0.32	0.08 (-0.07 to 0.22)		-0.10	-0.26	0.05 (-0.12 to 0.21)
CO (L/min)		1.6	2.6	0.8 (-0.7 to 2.3)		-1.7	-1.2	0.3 (-0.6 to 1.8)
TPR (dyn.s.cm^-5^)		126	108	-42 (-179 to 77)		-128	-168	-42 (-131 to 43)

CO, cardiac output; d_max_, post-dose maximum values; d_min_, post-dose minimum values; dZ/dt, rate of change of the transthoracic impedance; HR, heart rate; PEP, pre-ejection period; QS2, total electromechanic systole; TPR, total peripheral resistance.

Medians for baseline (Day 1), roflumilast alone (R; Day 11) and roflumilast plus formoterol (R+F; Day 18) with the corresponding point and 95% confidence interval (CI) estimates for untransformed (U) morning pre-dose values, untransformed post-dose maximum and minimum values (d_max _and d_min_), and Day 1-matched (δ) post-dose maximum and minimum values.

**Figure 4:**
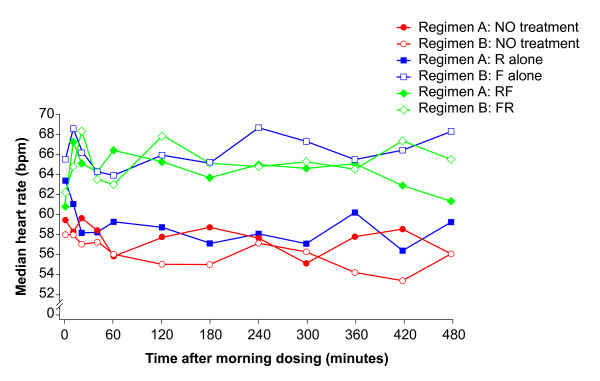
**Time course of the median heart rate throughout the main profiling days**. Regimen A: baseline Day 1 (NO treatment), Day 11 (R alone: roflumilast 500 μg once daily) and Day 18 (RF: roflumilast 500 μg once daily and formoterol 24 μg twice daily). Regimen B: baseline Day 1 (NO treatment), Day 8 (F alone: formoterol 24 μg twice daily) and Day 18 (FR: formoterol 24 μg twice daily and roflumilast 500 μg once daily).

**Figure 5:**
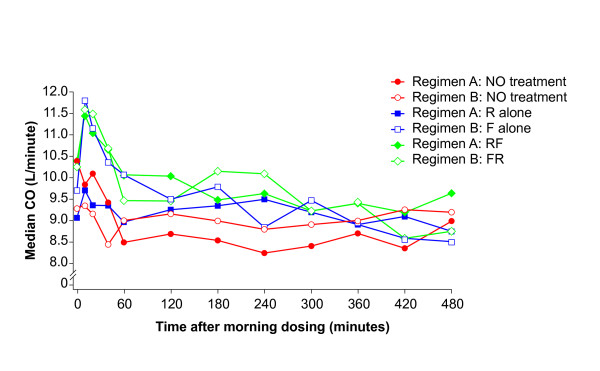
**Time course of the median cardiac output (CO) estimated by transthoracic impedance cardiography throughout the main profiling days**. Regimen A: baseline Day 1 (NO treatment), Day 11 (R alone: roflumilast 500 μg once daily) and Day 18 (RF: roflumilast 500 μg once daily and formoterol 24 μg twice daily). Regimen B: baseline Day 1 (NO treatment), Day 8 (F alone: formoterol 24 μg twice daily) and Day 18 (FR: formoterol 24 μg twice daily and roflumilast 500 μg once daily).

**Figure 6:**
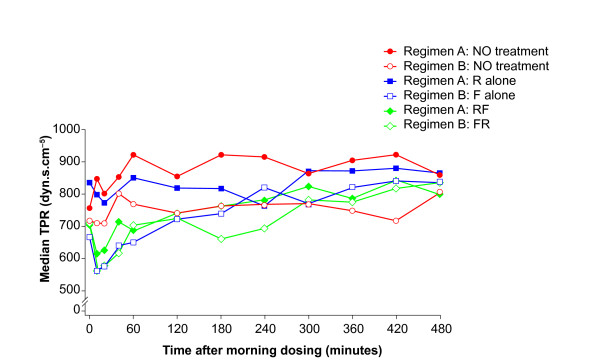
**Time course of the median total peripheral resistance (TPR) estimated by transthoracic impedance cardiography throughout the main profiling days**. Regimen A: baseline Day 1 (NO treatment), Day 11 (R alone: roflumilast 500 μg once daily) and Day 18 (RF: roflumilast 500 μg once daily and formoterol 24 μg twice daily). Regimen B: baseline Day 1 (NO treatment), Day 8 (F alone: formoterol 24 μg twice daily) and Day 18 (FR: formoterol 24 μg twice daily and roflumilast 500 μg once daily).

Roflumilast alone had little effect on cardiovascular function and there were only small changes in the ZCG/STI variables. Concomitant administration of formoterol resulted in a protracted rise in HR compared with roflumilast alone (Figure 4), which was associated with a rise in CO (Figure 5) and a drop in TPR within 10 minutes (Figure 6). Formoterol co-administration was also associated with an increase in dZ/dt_max_, particularly in the first hour after dosing, and an overall shortening of QS2 and PEP, which were mostly related to the rise in HR since they were less evident for the HR-corrected STIs (Table 4). These changes were small and within the normal physiological range.

#### Regimen B

The treatment medians for baseline Day 1, formoterol monotherapy alone (F; Day 8) and concomitant formoterol plus roflumilast (F+R; Day 18) and their contrasts are shown in Table 5. Compared with baseline, formoterol administration was associated with an increase in HR (Figure 4), a rise in CO (Figure 5) and a decrease in TPR (Figure 6), which were associated with an increased dZ/dt_max_, and an overall shortening in QS2 and PEP. These effects were clearly apparent on Day 8 and were not amplified by the concomitant administration of roflumilast.

**Table 5:** Pharmacodynamic measures - Regimen B: treatment medians and treatment contrasts

Untransformed pre-dose morning values								
	**Median**			**Point estimate (95% CI)**				

**Variable**	**Day 1**	**Day 8 (F)**	**Day 18 (F+R)**	**Day 8-Day 1**	**Day 18-Day 8**		**Day 18-Day 1**	

HR (bpm)	58	66	62	5 (2 to 9)	-1 (-4 to 3)		4 (2 to 7)	
PEP (ms)	105	97	98	-7 (-22 to 7)	3 (-9 to 17)		-5 (-19 to 20)	
QS2 (ms)	434	431	445	-4 (-18 to 9)	4 (-7 to 13)		2 (-13 to 16)	
dZ/dt (Ω/s)	1.81	1.94	1.82	0.06 (-0.07 to 0.19)	-0.08 (-0.37 to 0.07)		-0.03 (-0.30 to 0.16)	
CO (L/min)	9.7	9.7	10.3	0.7 (-0.9 to 2.0)	-0.5 (-1.7 to 0.6)		0.3 (-1.7 to 2.1)	
TPR (dyn.s.cm^-5^)	657	668	709	-18 (-198 to 70)	28 (-80 to 100)		-36 (-186 to 105)	

**Untransformed post-dose maximum and minimum values**								

	**d**_**max **_**(U)**				**d**_**min **_**(U)**			

	**Median**			**Point estimate (95% CI)**	**Median**			**Point estimate (95% CI)**

**Variable**	**Day 1**	**Day 8 (F)**	**Day 18 (F+R)**	**Day 8-Day 1**	**Day 1**	**Day 8 (F)**	**Day 18 (F+R)**	**Day 8-Day 1**

HR (bpm)	62	72	72	9 (5 to 11)	51	62	60	7 (3 to 10)
PEP (ms)	122	117	116	-4 (-14 to 2)	87	84	85	0 (-7 to 6)
QS2 (ms)	459	440	445	-14 (-27 to 4)	424	408	410	-13 (-22 to -3)
dZ/dt (Ω/s)	2.05	2.10	2.20	0.01 (-0.15 to 0.21)	1.53	1.64	1.60	0.03 (-0.13 to 0.17)
CO (L/min)	9.8	11.8	11.8	1.7 (0.6 to 2.6)	8.4	8.2	8.0	0.5 (-0.5 to 1.4)
TPR (dyn.s.cm^-5^)	870	882	865	-49 (-160 to 80)	655	561	556	-96 (-188 to -24)

**Day 1-matched post-dose maximum and minimum values**								

	**d**_**max **_**(δ)**				**d**_**min **_**(δ)**			

	**Median**			**Point estimate (95% CI)**	**Median**			**Point estimate (95% CI)**

**Variable**		**Day 8 (F)**	**Day 18 (F+R)**	**Day 18-Day 8**		**Day 8 (F)**	**Day 18 (F+R)**	**Day 18-Day 8**

HR (bpm)		16	14	-2 (-6 to 2)		-2	-3	-1 (-2 to 1)
PEP (ms)		25	21	3 (-19 to 4)		-26	-25	0 (-4 to 4)
QS2 (ms)		13	15	4 (-7 to 13)		-47	-37	8 (-1 to 15)
dZ/dt (Ω/s)		0.32	0.28	0.05 (-0.16 to 0.17)		-0.29	-0.37	-0.05 (-0.27 to 0.15)
CO (L/min)		2.9	3.3	0.0 (-1.0 to 1.5)		-0.6	-0.6	-0.4 (-1.3 to 0.6)
TPR (dyn.s.cm^-5^)		61	66	-18 (-95 to 73)		-221	-224	-42 (-107 to 35)

CO, cardiac output; d_max_, post-dose maximum values; d_min_, post-dose minimum values; dZ/dt, rate of change of the transthoracic impedance; HR, heart rate; PEP, pre-ejection period; QS2, total electromechanic systole; TPR, total peripheral resistance.

Medians for baseline (Day 1), formoterol alone (F; Day 8) and formoterol plus roflumilast (F+R; Day 18) with the corresponding point and 95% confidence interval (CI) estimates for untransformed (U) morning pre-dose values, untransformed post-dose maximum and minimum values (d_max _and d_min_), and Day 1-matched (δ) post-dose maximum and minimum values.

### Electrocardiograms

Both roflumilast and formoterol were associated with a slight increase in HR and, correspondingly, a decrease in the duration of the QT interval. QTc values, either uncorrected or corrected using Bazett's formula or Fridericia's formula, showed no clinically relevant changes. Categorical analyses of QT/QTc interval data did not reveal differences in the incidence of outliers between regimens, and the incidence of outliers was small. None of the subjects developed abnormal QT/QTc prolongation, and all QT/QTc intervals remained below 450 ms. No clinically relevant drug-related changes in PR or QRS intervals were observed. No clinically relevant regimen-related changes in ECG waveform morphology were detected.

### Serum glucose

With Regimen A, the median pre-dose serum glucose decreased from Day 1 (4.97 mmol/L) to Day 11 (4.75 mmol/L) and Day 18 (4.52 mmol/L). During Day 11 (roflumilast alone), glucose levels tended to remain slightly lower than pre-dose levels; in contrast, on Day 18, with concomitant formoterol plus roflumilast, there was a post-dose increase in serum glucose level that was close to the values throughout Day 1 (although the pre-dose levels had been lower).

With Regimen B, there was a slight post-dose increase in serum glucose throughout Day 8 compared with Day 1; addition of roflumilast did not appear to change this formoterol effect.

### Serum potassium

With Regimen A, the median pre-dose serum potassium level was lower during roflumilast administration (4.16 mmol/L) than at Day 1 (4.34 mmol/L) or during concomitant administration of the two agents (4.35 mmol/L) on Day 18. During Day 11, when roflumilast was administered alone, serum potassium levels remained slightly lower than levels throughout Day 1. On Day 18, with concomitant formoterol plus roflumilast, serum potassium levels tended to decrease from baseline, particularly between 1 and 6 hours after morning dosing.

With Regimen B, pre-dose concentrations were similar during formoterol administration (4.38 mmol/L) on Day 8 and during concomitant administration of formoterol and roflumilast (4.32 mmol/L) on Day 18 compared with Day 1 (4.48 mmol/L). On both Day 8 (formoterol) and Day 18 (concomitant administration), serum potassium levels tended to decrease from baseline throughout the course of the day.

### Safety

For Regimen A, during administration of roflumilast alone (Day 2 to Day 11), 7 subjects experienced 17 AEs; during subsequent concomitant administration of roflumilast and formoterol (Day 12 to Day 18), 8 subjects experienced 22 AEs. The most common AE was tremor, which was seen exclusively after addition of formoterol to roflumilast (11 of 22 AEs). Dizziness and myalgia were each reported on four occasions when roflumilast was administered alone; myalgia was also reported on four occasions when formoterol was added.

For Regimen B, during administration of formoterol alone (Day 2 to Day 8), no AEs were reported. Subsequently, during concomitant administration of formoterol and roflumilast (Day 9 to Day 18), 17 AEs were reported for 6 subjects; dizziness and headache were each reported on five occasions and tremor on two occasions.

All AEs were considered to be of mild (35/39 and 16/17 AEs reported with Regimens A and B, respectively) or moderate (4/39 and 1/17 AEs reported with Regimens A and B, respectively) intensity, and most were considered likely related to the investigational medication (35/39 and 14/17 AEs reported with Regimens A and B, respectively). There were no severe or serious AEs and no AEs led to premature discontinuation from the trial.

## Discussion

Roflumilast improves lung function in patients with COPD who are also treated with a long-acting bronchodilator [13]; accordingly, the concomitant administration of roflumilast and a β_2_-adrenoceptor agonist in patients with COPD is of interest. The present study investigated whether relevant drug-drug interactions might occur with the combination of oral roflumilast and inhaled formoterol. The steady-state plasma pharmacokinetics of roflumilast and its active metabolite roflumilast N-oxide were not altered by the addition of formoterol; similarly, the steady-state pharmacokinetics of formoterol were unaffected by the addition of roflumilast. Concomitant administration of inhaled formoterol resulted in changes that were less than 1% in peak concentration and exposure compared with roflumilast alone treatment Therefore, this co-administration is unlikely to result in clinically relevant interactions.

Cardiovascular effects were investigated by ZCG, which enabled measurement of STIs and allowed derivation of method-specific non-invasive estimates of systolic pump function [35,43]. These ZCG/STI methods are particularly sensitive to within-subject positive inotropic and vasodilatory cardiovascular changes, especially when chronotropic and inotropic reflexes are triggered by vasodilatation ('chrono-inodilatory' responses) [34,44]. Such measures are affected by changes in posture and food intake [45-47]. In all these measures the detectable effects were small. With formoterol alone there was a discrete trend towards a slightly higher HR, shorter PEP, increased CO and decreased TPR; these changes are likely to reflect a vasodilatory effect of formoterol [48,49]. Roflumilast had no effect on the ZCG/STI criteria and did not appear to potentiate the effects of formoterol.

Electrocardiographic investigations confirmed a slight increase in HR for formoterol, which was associated with an HR-dependent shortening of the QT interval with no change in HR-corrected QTc. Accordingly, there was no indication of a safety-relevant cardiovascular drug-drug interaction.

There was a slight decrease in fasting serum glucose level with roflumilast, which did not induce any hypoglycaemia. This effect was blunted when formoterol was administered concomitantly. Formoterol is known to increase serum glucose concentrations at supra-therapeutic levels [50]; however, at the 24 μg dose used in this study, there was only a slight increase in serum glucose level when formoterol was administered both alone and in combination with roflumilast. Supra-therapeutic doses of β_2_-agonists are also associated with decreased serum potassium concentrations [50,51]; in this study, a mild but consistent decrease was seen with formoterol, which was unaffected by concomitant roflumilast. A mild decrease in serum potassium level was also seen with roflumilast.

None of the other parameters examined (blood pressure, body temperature, clinical laboratory tests) showed safety-relevant changes that might be attributable to roflumilast, formoterol or their combination. The reported AEs were generally mild, with no severe or serious AEs, and mostly reflected the known properties of the investigated medications. There were some differences between the two regimens with regard to the incidence of tremor: tremor was reported 13 times when formoterol was added to roflumilast (11 times in Regimen A and twice in Regimen B), but was not observed when roflumilast or formoterol were administered alone (Regimens A and B respectively).

Although this study was performed in healthy men, results from clinical trials conducted in the roflumilast target population - patients with severe COPD, chronic bronchitis and a history of exacerbations - also support the absence of any relevant interaction between roflumilast and long-acting β_2_-adrenoreceptor agonists. The two 1-year roflumilast pivotal studies (M2-124 and M2-125) were conducted in 3091 patients, approximately half of whom were receiving concomitant β_2_-adrenoreceptor agonists [8]. In a pooled analysis of the two studies, roflumilast proved effective irrespective of β_2_-adrenoreceptor agonist use, and the overall pattern of AEs with or without concomitant β_2_-adrenoreceptor agonists was similar to that reported across all patients; there was no indication that roflumilast increased AEs associated with β_2_-adrenoreceptor agonists (such as tachycardia or cardiovascular events), and the co-administration of the two drugs did not increase the frequency of events associated with roflumilast alone [52]. Similar results were observed in a 6-month study (M2-127) conducted in patients with moderate to severe COPD receiving concomitant salmeterol [13]. Furthermore, a population pharmacokinetic analysis of roflumilast and roflumilast N-oxide [53] indicated that only a slight increase (12.6%) in tPDE4i is expected in patients with COPD compared with healthy individuals. The fact that during the clinical development of roflumilast the co-administration with long-acting β_2_-adrenoreceptor agonists was evaluated in patients with COPD for periods of up to 1 year also suggests that safety issues can be excluded, not only in the short term considered in this crossover study, but also in chronic use.

Given that β_2_-adrenoreceptor agonists can be used in combination with inhaled corticosteroids in the symptomatic treatment of severe COPD, concerns may arise regarding the addition of roflumilast to this therapeutic combination. Results from two 1-year randomised clinical trials (M2-111 and M2-112) in patients with severe and very severe COPD showed that the co-administration of inhaled corticosteroids does not affect the AE profile of roflumilast [55]. Furthermore, a drug-drug interaction study evaluating the effects of the co-administration of roflumilast and budesonide, a commonly used inhaled corticosteroid metabolised by CYP3A enzymes [54], revealed no relevant pharmacokinetic interactions and no alteration of the safety and tolerability profiles of either drug in healthy volunteers [24]. As mentioned earlier, roflumilast is metabolised by parallel CYP pathways, which suggests that specific CYP inducers or inhibitors are unlikely to alter its pharmacokinetic profile significantly. Although the combination of roflumilast, formoterol and inhaled corticosteroids has not been specifically investigated, based on these observations it is unlikely that the concomitant administration of the three drugs will cause relevant drug-drug interactions.

## Conclusions

In summary, the study results demonstrate that concomitant administration of oral roflumilast and inhaled formoterol under steady-state conditions does not affect the pharmacodynamics or pharmacokinetics of either drug. In particular, there was no evidence of a relevant pharmacodynamic interaction with regard to myocardial repolarisation or cardiac function in general. Moreover, the concomitant administration of roflumilast and formoterol did not negatively influence the safety profile of either drug.

## Competing interests

This study was sponsored by Nycomed GmbH (formerly ALTANA Pharma AG), Konstanz, Germany. CdM has received financial support for research and consulting services from Nycomed GmbH. NN and GL are employees of Nycomed GmbH, Konstanz, Germany.

## Authors' contributions

CdM served as a study investigator and was responsible for the acquisition of trial data. In addition he was involved in the study conception and design, in collaboration with GL. All three authors (CdM, GL and NN) contributed significantly to the analysis and interpretation of data; were involved in drafting the manuscript or revising it critically for important intellectual content; and gave final approval of the version to be published.

## Pre-publication history

The pre-publication history for this paper can be accessed here:

http://www.biomedcentral.com/1472-6904/11/7/prepub
